# A Semi-active Exoskeleton Based on EMGs Reduces Muscle Fatigue When Squatting

**DOI:** 10.3389/fnbot.2021.625479

**Published:** 2021-04-06

**Authors:** Zhuo Wang, Xinyu Wu, Yu Zhang, Chunjie Chen, Shoubin Liu, Yida Liu, Ansi Peng, Yue Ma

**Affiliations:** ^1^Chinese Academy of Sciences Key Laboratory of Human-Machine-Intelligence Synergic Systems, Shenzhen Institutes of Advanced Technology, Chinese Academy of Sciences, Shenzhen, China; ^2^Guangdong Provincial Key Lab of Robotics and Intelligent System, Shenzhen Institutes of Advanced Technology, Chinese Academy of Sciences, Shenzhen, China; ^3^School of Mechanical Engineering and Automation, Harbin Institute of Technology, Shenzhen, China; ^4^ShenZhen College of Advanced Technology, University of Chinese Academy of Sciences, Shenzhen, China

**Keywords:** semi-active exoskeleton, industrial exoskeleton, lower-limb exoskeleton, human-robot collaboration, EMG signal

## Abstract

In dynamic manufacturing and warehousing environments, the work scene made it impossible for workers to sit, so workers suffer from muscle fatigue of the lower limb caused by standing or squatting for a long period of time. In this paper, a semi-active exoskeleton used to reduce the muscle fatigue of the lower limb was designed and evaluated. (i) Background: The advantages and disadvantages of assistive exoskeletons developed for industrial purposes were introduced. (ii) Simulation: The process of squatting was simulated in the AnyBody.7.1 software, the result showed that muscle activity of the gluteus maximus, rectus femoris, vastus medialis, vastus lateralis, vastus intermedius, and erector spinae increased with increasing of knee flexion angle. (iii) Design: The exoskeleton was designed with three working modes: rigid-support mode, elastic-support mode and follow mode. Rigid-support mode was suitable for scenes where the squatting posture is stable, while elastic-support mode was beneficial for working environments where the height of squatting varied frequently.The working environments were identified intelligently based on the EMGs of the gluteus maximus, and quadriceps, and the motor was controlled to switch the working mode between rigid-support mode and elastic-support mode. In follow mode, the exoskeleton moves freely with users without interfering with activities such as walking, ascending and descending stairs. (iv) Experiments: Three sets of experiments were conducted to evaluate the effect of exoskeleton. Experiment one was conducted to measure the surface electromyography signal (EMGs) in both condition of with and without exoskeleton, the root mean square of EMGs amplitude of soleus, vastus lateralis, vastus medialis, gastrocnemius, vastus intermedius, rectus femoris, gluteus maximus, and erector spinae were reduced by 98.5, 97.89, 80.09, 77.27, 96.73, 94.17, 70.71, and 36.32%, respectively, with the assistance of the exoskeleton. The purpose of experiment two was aimed to measure the plantar pressure with and without exoskeleton. With exoskeleton, the percentage of weight through subject's feet was reduced by 63.94, 64.52, and 65.61% respectively at 60°, 90°, and 120° of knee flexion angle, compared to the condition of without exoskeleton. Experiment three was purposed to measure the metabolic cost at a speed of 4 and 5 km/h with and without exoskeleton. Experiment results showed that the average additional metabolic cost introduced by exoskeleton was 2.525 and 2.85%. It indicated that the exoskeleton would not interfere with the movement of the wearer Seriously in follow mode. (v) Conclusion: The exoskeleton not only effectively reduced muscle fatigue, but also avoided interfering with the free movement of the wearer.

## 1. Introduction

Despite the on-going trend in automation and mechanization in the industry, many workers still suffer from work-related musculoskeletal disorders due to unnatural body postures (De Looze et al., 2016; Huang et al., 2019). For example, in dynamic manufacturing and warehousing environments, the work scene made it impossible for workers to seat, so workers suffered from muscle fatigue of the lower limb caused by standing or squatting for a long period of time. Exoskeleton was suggested as a potential method to reduce exposure to activities and avoid postures that increase the risk of knee injury (Reid et al., 2010). Meanwhile, exoskeleton was suitable for application in such a dynamic scenario, which was not only providing support for the wearer anywhere, but also would not affect the free movement of the wearer.

An exoskeleton could be defined as a wearable, external mechanical device that augmented the performance of an able-bodied wearer, and helped disabled people to retrieve some motion abilities (Dollar and Herr, 2008; Krut et al., 2010; Huang et al., 2018). Exoskeletons could be divided into active exoskeleton and (quasi) passive exoskeleton according to whether there was a power supply. An active exoskeleton was driven by one or more actuators (e.g., electrical motor, pneumatic artificial muscle and hydraulic cylinder), so active exoskeleton was able to provide larger assistance force (Bosch et al., 2016). For example, the ReWalk Personal 6.0 System (Esquenazi et al., 2012), which has been developed for spinal cord injured patients (Rupal et al., 2017), was actuated by DC motors at the hip and knee joints; Muscle Suit Power, which was able to provide up to 35.7 *kgf* assistive force, was actuated by four McKibben artificial muscles (Kobayashi, 2016); Ekso NR was actuated by hydraulic actuators and was designed to help patients to relearn to correctly stand and work after stroke (Bionics, 2015). However, the efficiency and operation range were negatively affected due to the introduction of the heavy actuators and external power supplies (van Dijk and Van der Kooij, 2014). A (quasi) passive exoskeleton was driven by any type of actuator, but rather applied elastic materials, springs or dampers to store energy harvested by human motion and to use this as required to support a posture or a motion (De Looze et al., 2016). LegX (6.2 *kg*) (Pillai et al., 2020) and the Chairless Chair (Spada et al., 2018) were passive exoskeletons, which were used to reduce the effort of muscles when the wearer was in a position (e.g., squatting and semi-squatting) and they wished to maintain the position for a long time. But the chairless chair required wearers to fix a position by crouching down into the required position and pushing a button. And it was easy to interfere with the movement of the wearer because the joint axes of the Chairless Chair and wearer did not coincide.

In this paper, a semi-active exoskeleton was developed. With respect to already existing devices (Collo et al., 2016), special emphasis was placed on its light weight as well as multi working modes. The weight of the semi-active exoskeleton developed by us was only 2.6 kg, which was only 42% of the weight of the LegX and 74% of the weight of the Chairless Chair. Meanwhile, it was easy to switch among three working modes: rigid-support mod, elastic-support mode and follow mode. In rigid-support mode, the weight of the wearer was supported by the exoskeleton like a chair, instead of just relying on the legs when the wearer was squatting and semi-squatting. Meanwhile, the locking angle was adjusted easily by ratchet and pawl according to the flexion angle (0°~135°) of the knee joint. Elastic-support mode was beneficial for working environments where the height of squatting varied frequently, which provided assist force depending on the deformation of the torsion spring. In follow mode, the passive exoskeleton moved simultaneously with the legs of the wearer.

The main structure of this paper was as follows: the process and results of simulating squat were demonstrated in detail in section 2. The design of the exoskeleton was described in section 3. The experiment and result were presented in section 4. The exoskeleton was discussed in section 5.

## 2. Simulation

**Figure 1:**
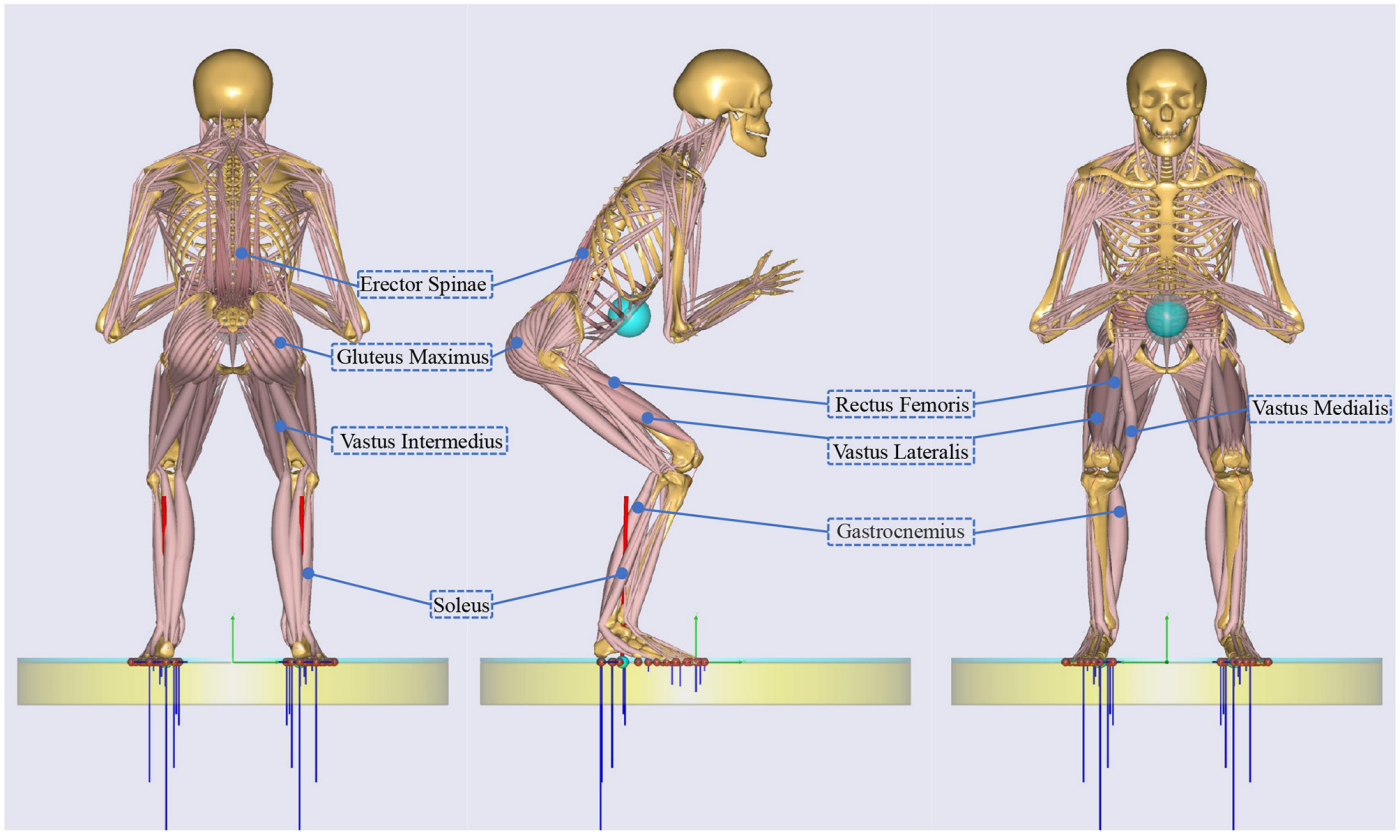
The main muscles involved in squatting.

Generally speaking, the locomotion system of humans was composed of skeletons, joints and muscles. The force generated by muscle contraction drove the skeleton to rotate around the joint (Nordin and Frankel, 2001; Neumann, 2013). Erector spinae, quadriceps femoris, gluteus maximus, and triceps surae are stretched when squatting, as shown in the Figure 1. Among them, quadriceps femoris included rectus femoris, vastus medialis, vastus medialis and vastus intermedius, triceps surae consisted of gastrocnemius and soleus. The force generated by stretched erector spinae compensated gravity to maintain a semi-squatting posture.

AnyBody (AnyBody.7.1, AnyBody Technology A/S, Aalborg, Denmark) was capable of analyzing the musculoskeletal system of humans (Damsgaard et al., 2006). To analyze the muscle activity of erector spinae, quadriceps femoris, gluteus maximus and triceps surae during squatting, the process of squatting was simulated in the AnyBody software. The musculoskeletal modeling and simulation of squatting were based on the demo provided by AnyBody Technology (Wu et al., 2020). The parameters in the musculoskeletal model were modified according to the subjects' body (De Roeck et al., 2020): The time period of a squat cycle and frames per second for simulation were set to 3 s and 30, respectively, minimum and maximum knee flexion angle of a squat cycle were set as 10° and 135°, squat distance between toe medial nodes shoulder width ratio and squat angle foot rotation were set to 1.6 and 5° separately.

**Figure 2:**
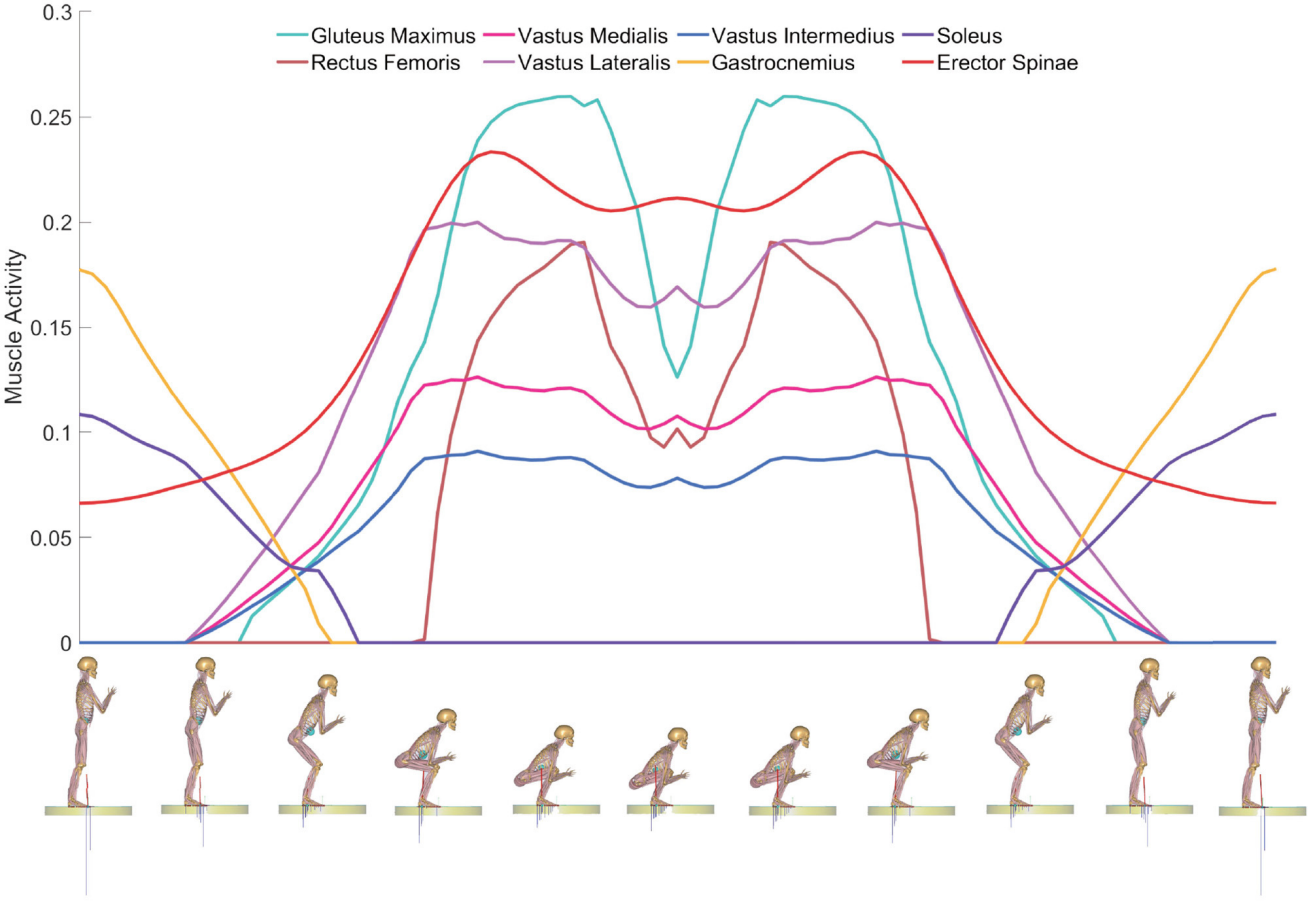
Changes in muscle activity of a squat cycle. The vertical and horizontal coordinates represent muscle activity and a squat cycle, respectively.

Changes in muscle activity of a squat cycle were shown in Figure 2. As the knee flexion angle gradually increased from 10° to 135°, the muscle activity of the gluteus maximus, rectus femoris, vastus medialis, vastus lateralis, vastus intermedius, and erector spinae first increased and then decreased, while the muscle activity of gastrocnemius and soleus decreased. The muscle activity of the gluteus maximus, rectus femoris, vastus medialis, vastus lateralis, vastus intermedius and erector spinae reached the maximum of 0.2598, 0.1904, 0.1264, 0.2, 0.091, and 0.2334, respectively, at the knee flexion angle of 115, 118, 54, 54, 96, and 52°. The higher the muscle activity value, the more intense the muscle contraction. So the function of the semi-active exoskeleton was aimed to reduce the muscle activity involved when squatting.

## 3. Design of the Exoskeleton

The assistive device presented in this work was used to provide support when the wearer was in a semi-squatting position for a long time and not interfere with movement when walking. So the special design was placed on its light weight as well as multi working modes.

### 3.1. System Overview

**Figure 3:**
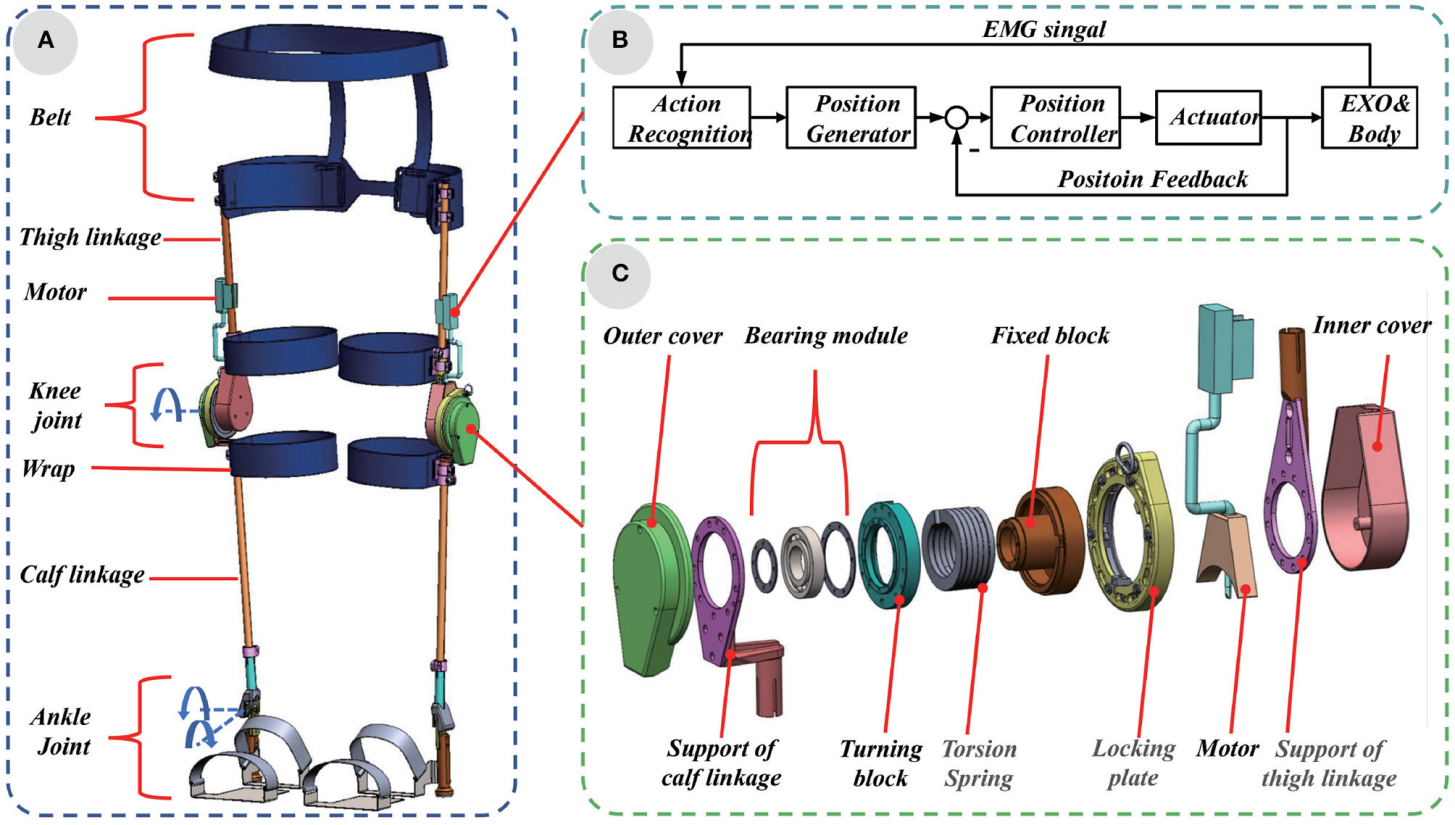
**(A)** System overview and composition of the semi-active exoskeleton. **(B)** The control system diagram based on EMGs. **(C)** The structure of the lockable knee joint.

As depicted in Figure 3A, the semi-active exoskeleton was mainly composed of belt, thigh linkage, motor, knee joint, wrap, calf linkage, and ankle joint. (i) the belt was adopted to fix the exoskeleton on the waist of the wearer and at transferred the weight of the body to the exoskeleton when squatting; (ii) thigh linkage and calf linkage made of carbon were tied on the lower limb by wraps and connected to a lockable knee joint; (iii) the knee joint could be locked when squatting and free to rotate within the range of 0° and 135° during walking; (iv) the ankle joint had two degrees of freedom, so it was able to rotate in sagittal plane and coronal plane. The total weight of our exoskeleton is 2,067 g. The weight of each component was given in Table 1.

**Table 1:** The weight of each component of our exoskeleton.

**Component**	**Mass (g)**	**Number**
Belt	305	1
Thigh linkage	91	2
Motor	205	2
Knee joint	326	2
Wrap	46	4
Calf linkage	33	2
Ankle joint	134	2
Total weight	2,067	-

The lockable knee joint was the main component, whose structure was shown in Figure 3C. The locking plate was adopted to adjust the angle of lockable knee joint according to the squatting position of wearer. In follow mode, the angle of the locking plate was adjusted to 0°, and the exoskeleton moves with the wearer without interfering with the daily activities such as normal walking, ascending and descending stairs and other daily activities. In the rigid-support mode, the angle of the locking plate was adjusted to the same angle as the angle of knee joint in any squatting position, the exoskeleton provided a comfortable support for the wearer. The linear motor was used to switch between rigid-support and elastic-support mode. When the height of the wearer's squatting posture changed frequently, the standard deviation of the EMGs of the gluteus maximus and quadriceps muscles increased more than 145 and 385 mv. Before calculating the standard deviation of the EMGs, which were filtered through 10~500*Hz* band pass filter and 50*Hz* notch filter (Neumann, 2013). When the standard deviation of the EMGs of the gluteus maximus and quadriceps muscle was greater than 145 and 385 mv, the linear motor was controlled to expand to limit the rotation of one end of the torsion spring. The exoskeleton worked in an elastic-support mode. Otherwise, the linear motor was controlled to contract, the torsion spring was not restricted. The exoskeleton worked in a rigid-support mode. At different squatting heights, the torsion spring was rotated at different angles, and assistance force was provided by the exoskeleton, the control system diagram was shown in Figure 3B.

### 3.2. Design

Theoretically speaking, it would be beneficial to improve assistance effect and comfort if the degree of freedom (DOF) of the exoskeleton was consistent with the DOF of the lower limbs of the human body. In general, the human lower limb could be taken as a structure with 7 DOFs. Three rotational DOFs at the hip, one rotation DOF at the knee, and three rotation DOFs at the ankle (Dollar and Herr, 2008). But the motion of squat mainly involved the flexions and extensions of the hip, knee, and ankle joints. In order to optimize the overall mechanical structure, knee joint capable of flexion and extension and ankle joint capable of flexion and extension, abduction and adduction were designed in the exoskeleton. The range of motion (ROM) of knee joint and ankle joint of the exoskeleton are set to match the ROM of humans during squatting. They are given in detail in Table 2. The ROM of exoskeleton was set between ROM at work and maximum ROM of human, which could protect the wearer's joint movement without interfering with the wearer's movement. At the same time, mechanical limit devices were designed at the end of the ROM of the knee and ankle joints to ensure the safety of the wearer.

**Table 2:** Range of motion of knee joint and ankle joint of exoskeleton.

**Joint**	**Motion**	**ROM at work(^**°**^)**	**Maximum ROM of human(^**°**^)**	**ROM of exoskeleton(^**°**^)**
Knee joint	Flexion	100	145	120
	Extension	0	0	0
Ankle joint	Dorsiflexion	20	50	30
	Plantarflexion	7	30	30
	Abduction	10	30	15
	Adduction	10	30	15

According to the result of squatting simulation in the AnyBody software, the muscle activity of quadriceps femoris and gluteus maximus increased. The belt and wrap made of nylon, were fixed on the hip area and the quadriceps area of the thigh. They functioned to the exoskeleton to the wearer's lower limb and transmitted the weight of the wearer to the supporting linkage of the thigh.

The support linkages of thigh and calf, which were made of carbon fiber, were connected together by a lockable knee joint. They were the main frame of the exoskeleton. The structure of the exoskeleton was designed for people with heights from 1.5 to 1.85 *m*. According to human dimensions, the adjustable ranges of thigh length, calf length and ankle height were set at 380 ~ 550 *mm*, 300 ~ 450 *mm*, and 60 ~ 80 *mm*, respectively.

The structure of the lockable knee joint was shown in Figure 3C. The lockable knee joint was composed of outer cover, bearing module, turning block, torsion spring, fixed block, locking plate, motor, and inner cover. The outer cover and inner cover were made of resin materials to protect the internal structure of the lockable knee joint. The axis of support of calf linkage, bearing module and turning block were fixed together with screws to coincide, while the axis of torsion spring, fixed block, locking plate, motor. and support of thigh linkage coincide. The turning block and fixed block, which were made of GCr15, were connected together with bearing components, so the support of calf and support of thigh could rotate coincidently. The motor was installed on the support of the thigh linkage with screws.

**Figure 4:**
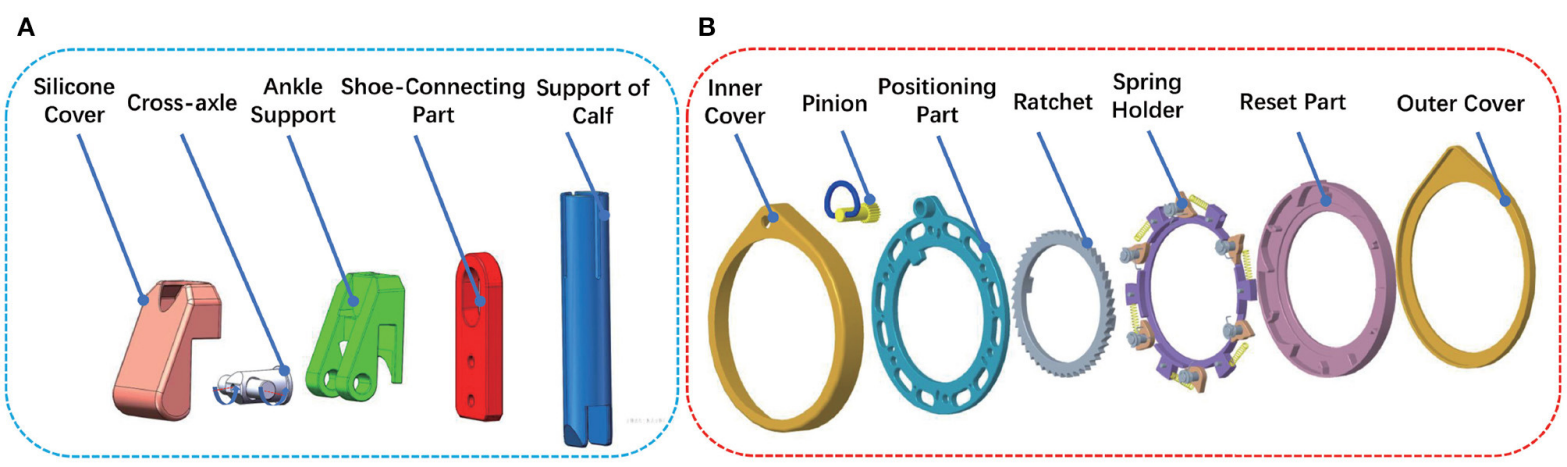
The structure of ankle joint **(A)** and locking plate **(B)**.

As depicted in Figure 4B. The locking plate was assembled by inner cover, pinion, positioning part, ratchet, spring holder, reset part, and outer cover. The inner cover and outer cover were made of resin materials, the pinion, positioning part, ratchet, and spring holder were made of Gcr15 and the reset part was made of aluminum alloy. Gear teeth were machined on the reset part, which was meshed with the pinion. Springs, torsional springs and pawls were installed in the spring holder. The engagement of the ratchet and the pawl ensure the positioning plate not only rotates in one direction around spring the holder. The rotation angle depended on the bosses of the inner ring of the ratchet and turning block. Adjusting the engagement of pinion and gear teeth on the reset part allows the ratchet to rotate in reverse.

The spring in the lockable knee joint was a customized torsional spring. The torsional spring was made of 60Si2MnA and it's cross-section was rectangle, which was the long side in the radial direction and the short side in the axial direction. The stiffness of the torsional spring was 0.8 *N*.*m*/°, which was calculated by Equation (1).

(1) $$\begin{array}{l} C_{T}=\frac{Ea^{3}b}{12\times 180D_{2}n} \end{array}$$

where *E* represented the elastic modulus of 60Si2MnA with a value of 206 *GPa*; *a* and *b* were the length and width of section of spring steel wire, whose values were 8 and 3.6 *mm*, respectively; *D*_2_ and *n* represented the mean diameter and number of coils, whose values were 40 *mm* and 5.5, respectively. According to the wearer's weight and applicable scenarios, torsion springs with different initial angles could be adjusted, so that the best elastic support can be obtained. One end of the torsion spring was fixed on the turning block. The torsion angle of the torsion spring was 0° when the knee joint was extended. However, the free end was restricted by motor, the torsion spring was twisted during the flexion of the knee joint. According to Equation (2), the energy storage of the torsion spring could be calculated.

(2) $$\begin{array}{l} E^{{\;}^{\prime }}=\int_{0}^{\theta }C_{T}\theta d\theta \end{array}$$

where *E*′ represented the energy storage of the torsion spring, θ was the torsion angle of spring, the physical quantity represented by *C*_*T*_ was the same as that in Equation (1). The energy was released to augment the muscle during knee extension.

**Figure 5:**
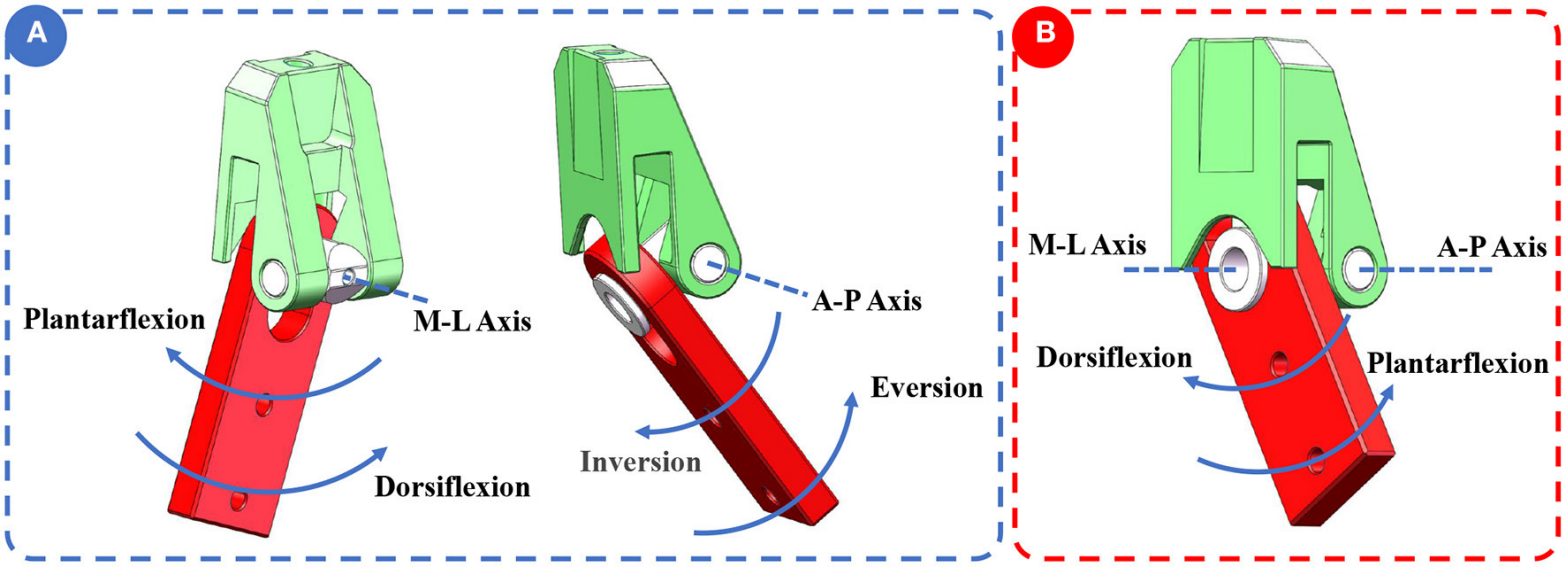
The working principle of ankle joint. **(A)** There were two degrees of freedom in ankle joint: plantarflexion/dorsiflexion, inversion/eversion. **(B)** Inversion and eversion were restricted.

The structure of the lockable ankle joint was depicted in Figure 4A. The lockable joint was mainly composed of silicon cover, cross-axle, ankle support, shoe-connecting part, and support of calf. The ankle support and shoe-connecting part were connected by cross-axle, allowing the shoe-connecting part to rotate in two directions around ankle support. In addition, cross-axles can slide slightly up and down along the chute on the shoe-connecting part. When the wearer's foot was off the ground, the shoe-connecting part slides down relative to cross-axle, which contributed to the dorsiflexion and plantarflexion, eversion and inversion, as shown in Figure 5A. When the wearer's feet were on the ground, especially in a squatting position, the shoe-connecting part slid upward relatively to cross-axle, and the shoe-connecting part was restricted by the locking slot on the ankle support, which restricted eversion and inversion movement, as shown in Figure 5B. Since the ankle joint had no eversion and inversion movement, the exoskeleton can provide stable support.

## 4. Experiments

In order to evaluate the performance of the exoskeleton, three different experiments were conducted. In the first experiment, the EMGs of muscles related to squatting were measured in both conditions of with and without exoskeleton, to validify whether the exoskeleton can relieve muscle fatigue. The second experiment was to test the effective support that the exoskeleton can provide under different weights and squatting positions. Experiment three was used to measure the additional metabolic cost introduced by the exoskeleton to wearer in follow mode. All experiments were performed in a laboratory at a stable indoor temperature of 26^*o*^*C*. Before each experiment, subjects were instructed to get familiar with exoskeleton and exoskeleton was adjusted accordingly.

Four healthy subjects, with no leg diseases, volunteered to participate in three experiments. The contents and impacts of experiments were informed in detail to them and their contents were obtained. The human body's characteristics dimensions of subjects were detailed in Table 3.

**Table 3:** The human body's characteristics dimensions of subjects.

**Subject**	**1**	**2**	**3**	**4**
Gender	Woman	Man	Man	Man
Stature (*cm*)	160	176	177	183
Weight (*kg*)	48	67.5	80	70
Thigh length (*mm*)	435	483	480	540
Calf length (*mm*)	340	400	418	440
Ankle height (*mm*)	52	81	80	83
Shoes size (*cm*)	23	26	26.5	26.5

Each experiment was divided into an experimental group and a control group. The experimental group was required to wear the exoskeleton, while the control group was not required to wear the exoskeleton. In order to avoid the influence of human muscle fatigue on the experiment, both the experimental group and the control group were conducted for 2 days, respectively. The experiment procedures of the control group and the experimental group were the same.

### 4.1. EMGs Measurement

Surface myoelectric signal analysis has been proved effective for assessing the electrical manifestations of localized muscle fatigue (Pi et al., 2006). In order to accurately evaluate the effect of exoskeleton in reducing muscle fatigue, the EMGs of the muscle related to squatting with and without exoskeleton were measured in this experiment.

**Figure 6:**
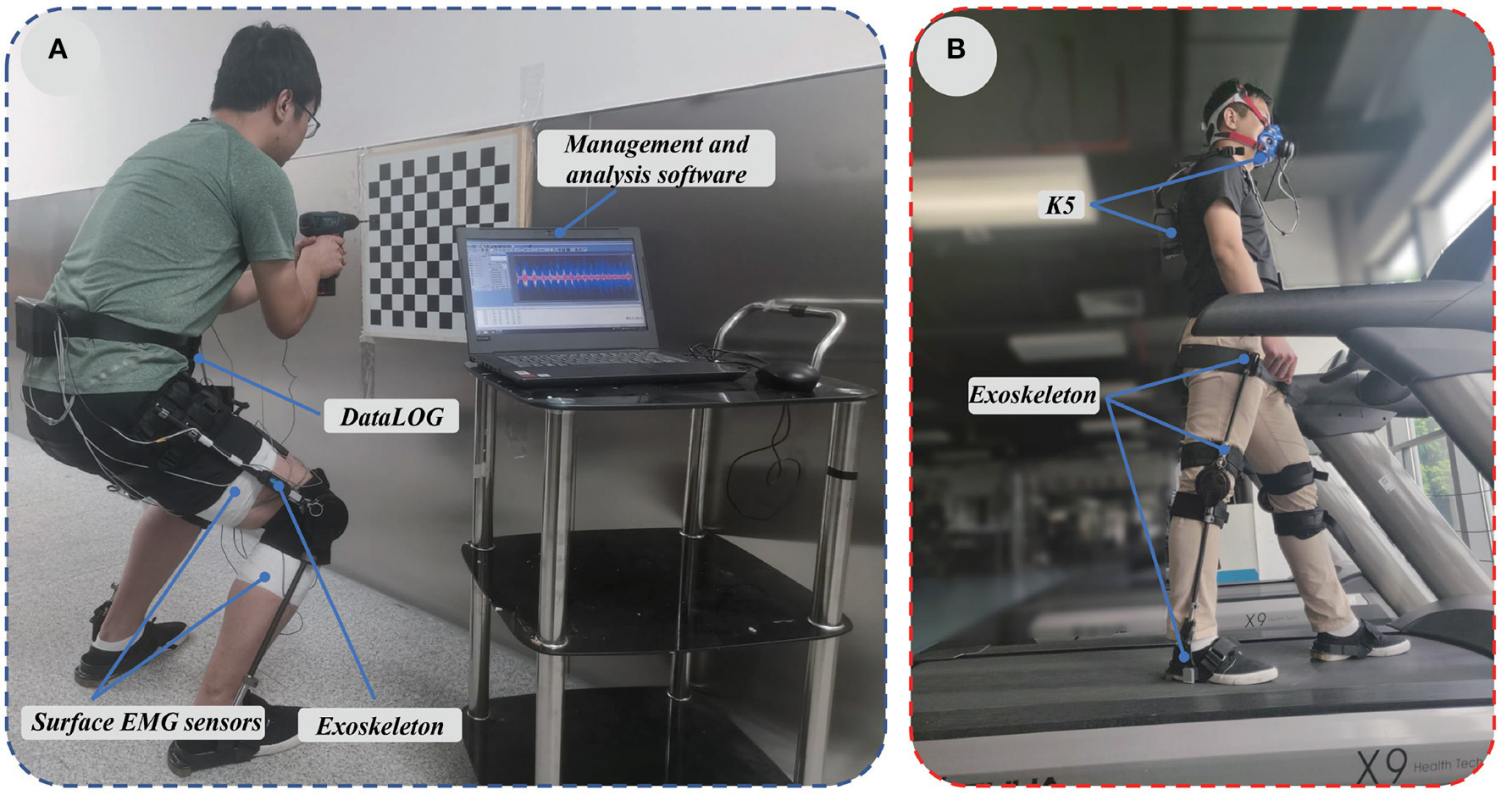
**(A)** EMGs of the muscle related to squatting with and without exoskeleton were measured in this experiment. **(B)** The metabolic cost with and without exoskeleton was measured.

First of all, the skin was shaved, scrubbed, and cleaned with alcohol swab before the surface ENG sensors were applied (Bosch et al., 2016). Secondly, eight surface EMG sensors (SX230, Biometrics Limited, UK) were attached by EMG Sensor Tapes (T350) to the skins of soleus, vastus lateralis, vastus medialis, gastrocnemius, vastus intermedius, rectus femoris, gluteus maximus, and erector spinae. And then surface EMG Sensors were wrapped around the subject's limb by gauze to prevent it from falling and the reference electrode (R306), was wrapped on the wrist joint by elastic bands. Next, the surface EMG sensors and reference electrode were connected to an eight-channel DataLOG (MWX8), which transmitted real-time data to computers via Bluetooth Wireless link. Finally, subjects were instructed to use an electric drill to install screws on the board that simulating assembly works, whose process lasted for 2 min. Drilling different holes in the horizontal direction of the board to simulate the scene of squatting height stability (worked in a rigid-support mode), while drilling different holes in the vertical direction of the board to simulate the scene of squatting height varied (worked in an elastic-support mode). According to the simulation results in AnyBody software, the muscle activity was higher when the knee flexion angle was 120°, so the surface EMGs were recorded at 500*Hz* when the knee flexion angle was 120±10°. The experiment setup was shown in Figure 6A.

**Figure 7:**
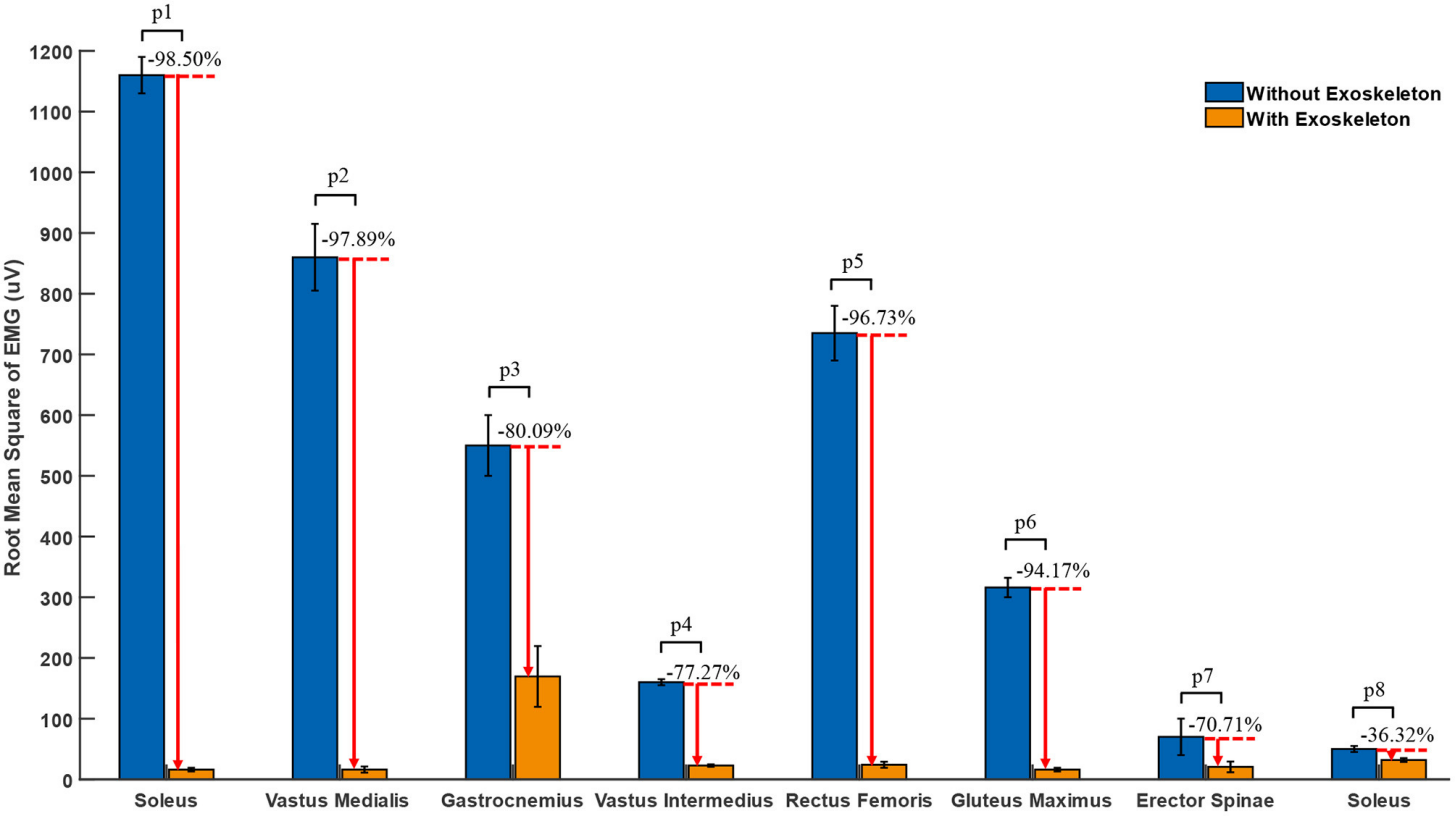
The RMS of the EMGs of the knee joint at 120 degree flexion angle. p1, p2, p3, p4, p5, p6, p7, and p8 are the result of two-side *t*-tests, which are 0.0002, 0.0014, 0.0044, 0.0014, 0.0013, 0.0004, 0.1112, and 0.0131, respectively.

Average rectified value (ARV), integrated electromyogram (iEMG) and root mean square (RMS) of EMGs showed an upward trend during muscles fatigue (Viitasalo and Komi, 1977; Madeleine et al., 2002). Changes in these values were usually related to muscle contraction, but RMS could better reflect muscle fatigue under the same muscle contraction state (Jiang et al., 2017). After EMGs were filtered through 10~500*Hz* band pass filter and 50 Hz notch filter (Neumann, 2013), RMS was calculated according to Equation (3).

(3) $$\begin{array}{l} RMS=\sqrt{\frac{1}{N}\overset{N}{\underset{i=1}{\sum }}EMG^{2}\left(i\right)} \end{array}$$

where RMS represented the root mean square of EMGs, *N* represented the number of EMGs collected within the sampling time, *EMG*(*i*) represented the amplitude of the i-th EMGs. The root mean square of EMGs amplitude of soleus, vastus lateralis, vastus medialis, gastrocnemius, vastus intermedius, rectus femoris, gluteus maximus, and erector spinae were reduced by 98.5, 97.89, 80.09, 77.27, 96.73, 94.17, 70.71, and 36.32%, respectively with the assistance of the exoskeleton, which was shown in Figure 7. We can conclude that the exoskeleton was able to reduce muscle fatigue effectively.

### 4.2. Plantar Pressure Measurement

The working principle of the exoskeleton to reduce muscle fatigue was that the weight of wearer was transmitted to the ground by the exoskeleton and the lower limbs, rather than just by the lower limbs of wearer. To measure the support force provided by exoskeleton under different weights and squatting positions, the foot pressure was measured in this experiment.

**Figure 8:**
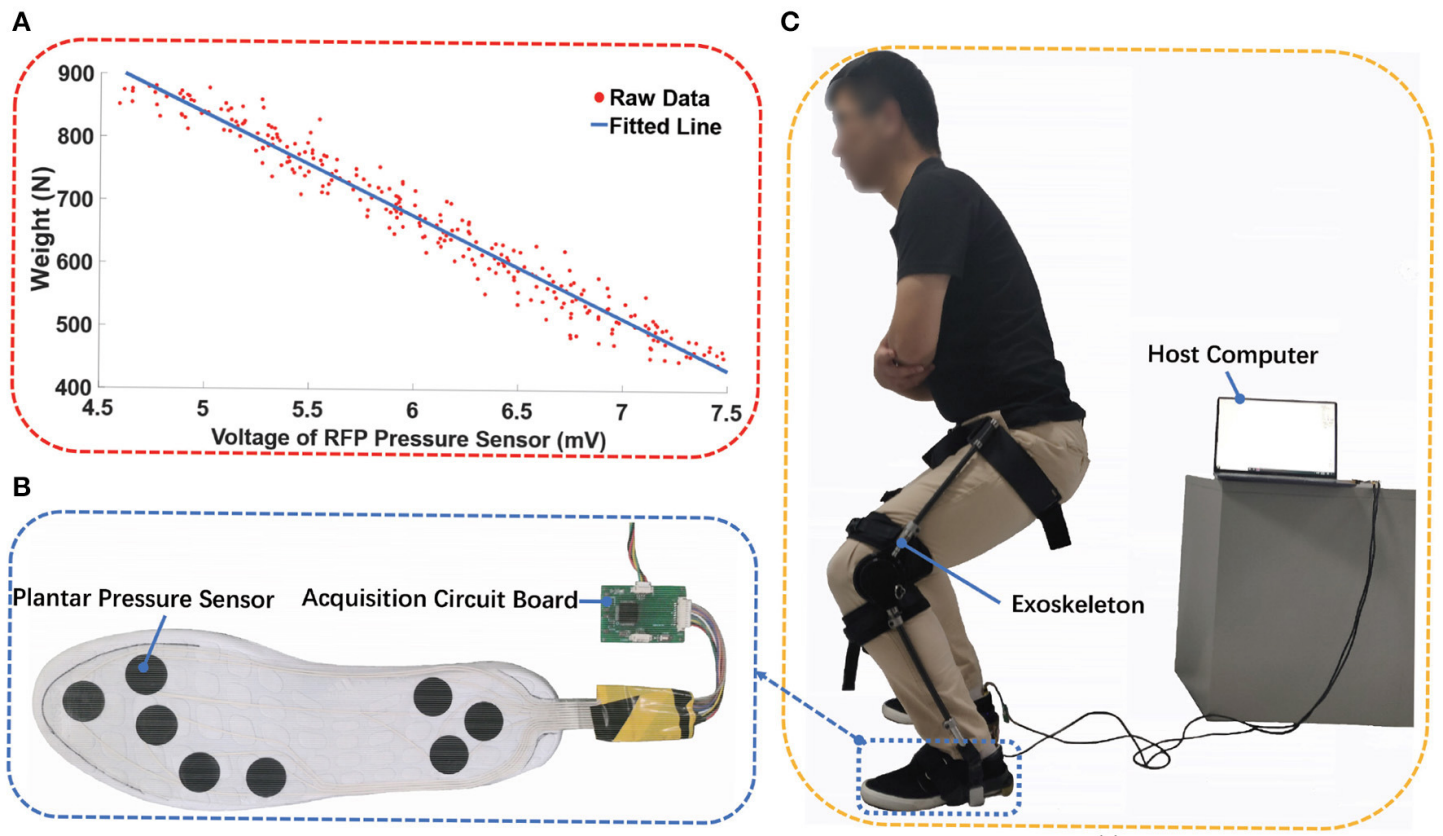
Plantar pressure measurement. **(A)** The splatting was the raw data from experiment and the blue line is fitted line, **(B)** the plantar sensor and data acquisition circuit board, **(C)** the foot pressure experiment was conducted.

Before the starting experiment, experiment acquisition system was designed, which was composed of two plantar pressure sensors (ZNX-01, Suzhou Leanstar Electronic Technology Co., ltd., China) and bluean acquisition circuit board, which communicated with XCOM software (V2.2, download from http://www.openedv.com/) through a serial port at speed of 460800 bps, as shown in Figure 8B. To analyze the relationship between the weight and voltage of RFP pressure sensor, the subjects were required to carry additional loads, whose weight was increased from 0 to 5 kg with an increment of 0.5*kg* each time. The subjects were instructed to stand 30 s at different loads. As shown in Figure 8A, the splattering points were the average value of voltage of RFP pressure sensor and weight, the blue line was the fitted line as expressed by Equation (4).

(4) $$\begin{array}{l} G=-162.3U+1649.7 \end{array}$$

where the G was the weight (the sum of weight of subject and additional load) of the subject and U represented the voltage value.

At first, the weight and voltage of RFP pressure sensor were recorded when the subjects were instructed to flex the knee without exoskeleton at 60°, 90°, and 120°, respectively. Each test lasted for 1 min and a 5-min rest was set. After the exoskeleton was adjusted to fit the subject, the same experiment was conducted again, as shown in Figure 8C. The exoskeleton worked in a rigid-support mode.

**Figure 9:**
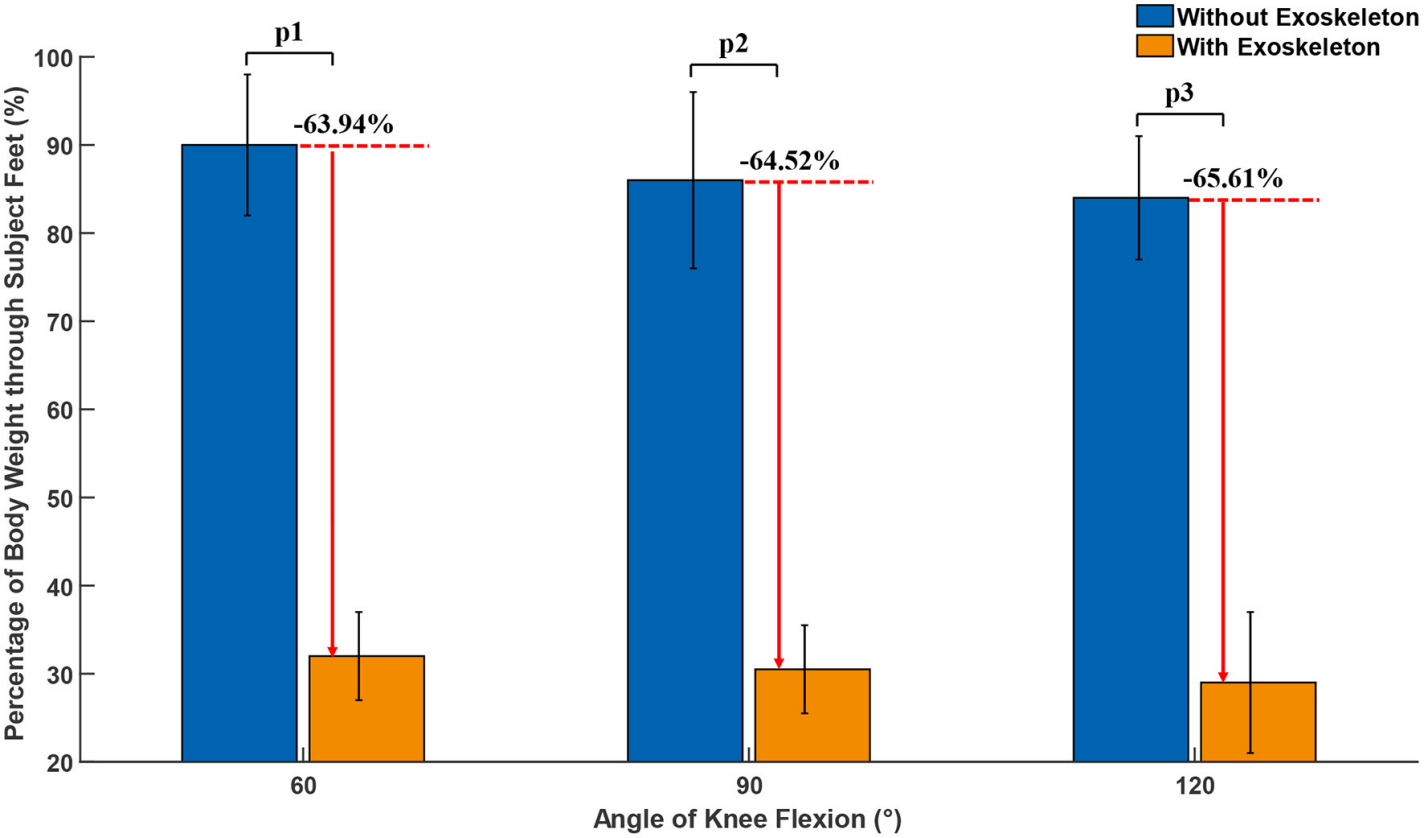
Percentage of body weight through subject Feet at 60°, 90°, and 120° of knee flexion angle. p1, p2, and p3 are the result of two-side *t*-tests, which are 0.0018, 0.0036, and 0.0009, respectively.

To eliminate the influence of unstable squatting posture, the data of the first 15 s and the last 15 s were removed, and only the data in between were selected for analysis. After the data was filtered by median filter, the plantar force of both conditions were calculated according to Equation (4). Taking into account the differences of subject, the plantar force was normalized using the weight of the subject, as shown in Figure 9. The reason why the percentage of body weight through subject feet at 60°, 90°, and 120° of knee flexion angle was less than 100% was that there was a difference in the pressure distribution on the soles of the feet when standing and squatting. In particular, parts of weight were transmitted to ground through the areas of arches of the foot, on which no RFP pressure sensor was installed. Comparing the percentage of weight of both conditions, wearing an exoskeleton reduces the weight through the subject's feet by 63.94, 64.52, and 65.61%, respectively at 60, 90, and 120° of knee flexion angle. Moreover, the assistive force provided by the exoskeleton increased as the angle of knee flexion increased, which can be attributed to the increase in the effective contact area between the belt and the subject.

Table 4 showed a comparison between our exoskeleton and the Chairless Chair. The exoskeleton designed by us was only 2.1 kg, which was lighter than 3.5kg of the Chairless Chair. Meanwhile, only 29.3% of weight was supported by the subject's feet after wearing the exoskeleton, which was effectively the weight transmitted by the feet.

**Table 4:** Comparison with the chairless chair.

**Exoskeleton**	**Weight (kg)**	**Percentage of body weight through subject feet (%)**
Chairless Chair	3.5 (Spada et al., 2018)	33
This work	2.1	29.3

### 4.3. Metabolic Cost Measurement

In order to evaluate whether the exoskeleton interfered with the subjects' movement in the following mode, such as walking between the workbenches of the assembly line. The wearable metabolic system (K5, COSMED, Italy) was adopted to measure the concentration and volume of the exhaled pulmonary gas, which are mainly carbon dioxide and oxygen (Chen et al., 2020). In experiment, the metabolic cost under the conditions of with and without exoskeleton were measured when the subject was walking at the speed of 4 and 5 km/h on a treadmill (SH-5918, ShuHua Sports Co., Ltd., China).

Each experiment lasted for 20 min and was divided into three stages, which as shown in Figure 6B. First, the subject was asked to maintain a standing position for 5 min in order to measure the standing cost; Then, the subject walked for 10 min to obtain the walking metabolic cost; Finally, the subject was also instructed to maintain a standing position for 5 min.

The method of calculating the net metabolic cost was subtracting the standing metabolic cost from the walking metabolic. Taking into the unstable metabolic cost at the beginning and the transition from standing to walking, the data from the 3rd to the 5th min, the 7th to the 13th min, and the 17th to the 20th min in each experiment are selected to calculated based on Equation (5) (Garby and Astrup, 1987).

(5) $$\begin{array}{l} \Delta H=c_{1}VO_{2}+c_{2}VCO_{2} \end{array}$$

where Δ*H* was the energy rate (*kJ*/*s*), coefficients *c*_1_ and *c*_2_ were 16.04 and 4.94*kJ*/*L*, respectively, the unit of *VO*_2_ and *VCO*_2_ was *L*/*s*. The result of metabolic cost measurement when the subject was walking at the speed of 4 and 5 km/h were shown in Table 5. The average additional metabolic cost introduced by exoskeleton was 2.525 and 2.85%. It means that the exoskeleton would not interfere with the movement of the wearer in follow mode.

**Table 5:** The result of metabolic cost measurement when the subject was walking at the speed of 4 and 5 km/h.

**Subject**	**4 km/h**			**5 km/h**		
	**WOE (W/kg)**	**WE (W/kg)**	**AMC (%)**	**WOE (W/kg)**	**WE (W/kg)**	**AMC (%)**
1 (female)	4.20	4.33	3.1	4.69	4.82	2.8
2 (male)	5.32	5.42	1.9	5.81	5.98	2.9
3 (male)	5.27	5.43	3.0	5.75	5.89	2.4
4 (male)	5.78	5.90	2.1%	5.82	6.01	3.3

*Where WOE, WE, and AMC were the abbreviation of without exoskeleton, with exoskeleton and additional metabolic cost, respectively*.

## 5. Discussion

A semi-active exoskeleton was designed in this work, used to reduce the muscle fatigue of the lower limb when squatting.

In order to analyze the muscle activity of each muscle in the process of squatting, so that the designed exoskeleton can effectively reduce muscle fatigue, the process of squatting was simulated in the AnyBody software. As the knee flexion angle gradually increased from 10° to 135°, the muscle activity of the gluteus maximus, rectus femoris, vastus medialis, vastus lateralis, vastus intermedius, and erector spinae first increased and then decreased, while the muscle activity of gastrocnemius and soleus decreased. Therefore, the exoskeleton was designed to provide assistance force for the gluteus maximus, rectus femoris, vastus medialis, vastus lateralis, vastus intermedius and erector spinae.

The exoskeleton was applicable for users from 150 to 185 *cm*, which has the degree of freedom of flexion and extension of knee joint, dorsiflexion, plantarflexion, eversion and inversion of ankle joint. Because its parts were made of carbon fiber and aluminum alloy, the total weight of the exoskeleton was only 2,067 g, which was only 33.9% of Legx and 60% of the Chairless Chair. It was easy to switch among three working modes: follow mode, rigid-support mode and elastic-support mode. In the follow mode, the angle of the locking plate was adjusted to 0°, and the assist device can follow the wearer's movement without interfering with the daily activities such as normal walking, upstairs and down-stairs and other daily activities. In the rigid-support mode, the angle of the locking plate was adjusted to the same angle as the angle of the knee joint in any squatting position. The assistive device provided comfortable support for the wearer. In the elastic-support mode, the energy is harvested by a torsion spring during knee-flexion and was released during knee-extension, which was used to reduce the moment required for the knee joint during knee-extension.

In order to evaluate the performance of the exoskeleton, three different experiments were conducted, respectively. In the first experiment, the EMGs of the muscles related to squatting with and without exoskeleton was measured, the result showed that the mean EMGs amplitude of soleus, vastus lateralis, vastus medialis, gastrocnemius, vastus intermedius, rectus femoris, gluteus maximus, and erector spinae were reduced by 98.5, 97.89, 80.09, 77.27, 96.73, 94.17, 70.71, and 36.32%, respectively, with the assistance of the exoskeleton. The second experiment was to test the effective support that the exoskeleton can provide under different weights and squatting positions. Compared to the percentage of weight through subject's feet without and with exoskeleton, it was reduced by 63.94, 64.52, and 65.61%, respectively, at 60°, 90°, and 120° of knee flexion angle. Experiment three was used to measure the additional metabolic cost brought by the exoskeleton to wearer in follow mode. The average additional metabolic cost introduced by exoskeleton was 2.525 and 2.85%. Each kilogram added to the foot increases energy expenditure 7–10% (Knapik et al., 2004), which means the average additional metabolic cost introduced by exoskeleton was less than that worn by safety shoes (the weight of the safety shoes was greater than 1 kg). Therefore, it means that the exoskeleton would not seriously interfere with the movement of the wearer in follow mode.

The exoskeleton not only effectively reduced muscle fatigue, but also did not interfere with the free movement of the wearer. In the future, the intelligent switcher will be designed, which will enable the exoskeleton to switch in three working modes intelligently according to the intention of wearer.

## Data Availability Statement

The original contributions generated for the study are included in the article/supplementary material, further inquiries can be directed to the corresponding author/s.

## Ethics Statement

The studies involving human participants were reviewed and approved by the Medical Ethics Committee of Shenzhen Institutes of Advanced Technology. The participants provided their written informed consent to participate in this study.

## Author Contributions

ZW and XW: conceptualization and writing–original draft preparation. ZW and AP: methodology. ZW and YZ: software. ZW, YZ, and AP: validation. ZW: formal analysis and data curation. AP and CC: investigation. CC and SL: resources. YL and YM: writing–review and editing. SL: visualization. CC: supervision. XW and CC: project administration and funding acquisition. All authors have read and agreed to the published version of the manuscript.

## Conflict of Interest

The authors declare that the research was conducted in the absence of any commercial or financial relationships that could be construed as a potential conflict of interest. The reviewer HC declared a shared affiliation, with no collaboration, with the authors ZW, YZ, SL to the handling editor at the time of the review.
